# SOCIAL ISOLATION, LONELINESS, AND MEN'S HEALTH

**DOI:** 10.1093/geroni/igz038.781

**Published:** 2019-11-08

**Authors:** Harry O Taylor

**Affiliations:** Washington University in St. Louis, St. Louis, Missouri, United States

## Abstract

Strong and fulfilling relationships are important components of men’s health and well-being across the life course; however, social isolation and loneliness are important but under-assessed conditions among older men. This is important to note because older men often subscribe to common masculinity themes regarding independence and self-sufficiency which places them at greater risk for social isolation and loneliness in comparison to older women. The purpose of this presentation is to review the social isolation and loneliness literature specifically among older men by 1) discussing gender differences, and the potential mechanisms behind these differences, in social isolation and loneliness, 2) examining health, behavioral and physiological effects of social isolation and loneliness specifically among older men, and 3) providing future research directions for understanding social isolation and loneliness among older men including understanding social isolation and loneliness and their associative outcomes among diverse samples of older men.

